# Understanding a constellation of eight COVID-19 disease prevention behaviours using the COM-B model and the theoretical domains framework: a qualitative study using the behaviour change wheel

**DOI:** 10.3389/fpubh.2023.1130875

**Published:** 2023-07-05

**Authors:** Angel M. Chater, Phoebe Brook-Rowland, Foyeke Tolani, Emily Christopher, Jo Hart, Lucie M. T. Byrne-Davis, Abby Moffat, Gillian W. Shorter, Tracy Epton, Atiya Kamal, Daryl B. O’Connor, Eleanor Whittaker, Lesley J. M. Lewis, Emily McBride, Vivien Swanson, Madelynne A. Arden

**Affiliations:** ^1^Centre for Health, Wellbeing and Behaviour Change, Institute for Sport and Physical Activity Research, University of Bedfordshire, Bedford, United Kingdom; ^2^Centre for Behaviour Change, University College London, London, United Kingdom; ^3^Bedford Borough, Central Bedfordshire and Milton Keynes Councils’ Shared Public Health Services, Bedford, United Kingdom; ^4^School of Medical Sciences, University of Manchester, Manchester, United Kingdom; ^5^Drug and Alcohol Research Network, School of Psychology, Queen’s University Belfast, Belfast, United Kingdom; ^6^Manchester Centre for Health Psychology, University of Manchester, Manchester, United Kingdom; ^7^School of Social Sciences, Birmingham City University, Birmingham, United Kingdom; ^8^Laboratory for Stress and Health Research, University of Leeds, Leeds, United Kingdom; ^9^North Yorkshire County Council, Northallerton, North Yorkshire, United Kingdom; ^10^Behavioural Science Unit, Public Health, Somerset County Council, Taunton, United Kingdom; ^11^Department of Behavioural Science and Health, University College London, London, United Kingdom; ^12^Psychology Division, University of Stirling, Stirling, United Kingdom; ^13^Centre for Behavioural Science and Applied Psychology, Sheffield Hallam University, Sheffield, United Kingdom

**Keywords:** COVID-19, behaviour change, hand hygiene, face covering, physical-distancing, testing, vaccination, COM-B

## Abstract

**Background:**

The use of behavioural science and behaviour change within local authorities and public health has supported healthful change; as evidenced by its importance and contribution to reducing harm during the COVID-19 pandemic. It can provide valuable information to enable the creation of evidence-based intervention strategies, co-created with the people they are aimed at, in an effective and efficient manner.

**Aim:**

This study aimed to use the COM-B model to understand the Capability, Opportunity and Motivation of performing a constellation of eight COVID-19 disease prevention behaviours related to the slogans of ‘Hands, Face, Space, Fresh Air’; ‘Find, Isolate, Test, (FIT), and Vaccinate’ in those employed in workplaces identified as high risk for transmission of the SARS-CoV-2 (severe acute respiratory syndrome coronavirus 2) to support intervention development.

**Methods:**

This qualitative study recruited twenty-three participants (16 female, 7 male), who were interviewed from three environments (schools, care homes, warehouses) across three local authorities. Semi-structured interviews were analysed using thematic analysis.

**Findings:**

Ten core themes were identified inductively; (1) knowledge and skills, (2) regulating the behaviour, (3) willingness to act, (4) necessity and concerns, (5) emotional impact, (6) conducive environment, (7) societal influence, (8) no longer united against COVID-19, (9) credible leadership, and (10) inconsistent adherence to COVID-19 prevention behaviours. Themes were then deductively mapped to the COM-B model of behaviour change and the theoretical domains framework and a logic model using the behaviour change wheel (BCW) was produced to inform intervention design.

**Conclusion:**

This study offers a novel approach to analysis that has included eight behaviours within a single thematic analysis and COM-B diagnosis. This will enable local authorities to direct limited resources to overarching priorities. Of key importance, was the need for supportive and credible leadership, alongside developing interventions collaboratively with the target audience. COVID-19 has had an emotional toll on those interviewed, however, promoting the value of disease prevention behaviours, over and above their costs, can facilitate behaviour. Developing knowledge and skills, through education, training, marketing and modelling can further facilitate behaviour. This supports guidance produced by the British Psychological Society COVID-19 behavioural science and disease prevention taskforce.

## 1. Introduction

Health psychology is a discipline in behavioural science that can help to guide the development of effective interventions to enhance population health and wellbeing (1–3). To do this, a ‘problem’ needs to be defined in behavioural terms, followed by the generation of insight through a ‘behavioural diagnosis’ using the COM-B model, the hub of the Behaviour Change Wheel (4, 5). This COM-B analysis can identify issues of Capability (psychological and physical: e.g. knowledge and skills), Opportunity (physical and social: e.g. environmental factors and social influences) and Motivation (reflective and automatic: e.g. beliefs and emotion) that may influence Behaviour (hence COM-B: Capability, Opportunity, Motivation – Behaviour).

As of October 2022, in the United Kingdom there were 18,826,374 cases of people testing positive for COVID-19 on a first occasion, 1,312,947 re-infections and 183,579 deaths with COVID-19 on the death certificate (6). There is a constellation of eight core COVID-19 disease prevention behaviours, namely: (1) hand hygiene in the form of cleaning hands with soap and water and/or alcohol-based gel; (2) wearing face coverings to mitigate airborne transmission from close contact, (e.g., in work or public settings); (3) physical-distancing (also known as social-distancing; which in the United Kingdom promoted the maintenance of 2 m physical distance between individuals from differing households and/or ‘social bubbles’); (4) ensuring good ventilation through ventilation systems and/or opening windows/doors; (5) PCR (polymerase chain reaction) testing for COVID-19 at the onset of symptoms; (6) regular use of lateral flow device (LFDs) tests (also referred to as LFTs) for COVID-19 whilst asymptomatic; (7) self-isolation following a positive COVID-19 test or being identified as a close contact and (8) vaccination uptake (7). This constellation of behaviours falls under the slogans of ‘Hands, Face, Space, Fresh Air’; ‘Find, Isolate, Test, (FIT), and Vaccinate’.

At various points in the pandemic, the United Kingdom government and devolved nations’ public health agencies implemented country-specific intervention strategies, including guidelines (e.g., physical-distancing), regulation/legislation (e.g., wearing face coverings in indoor public places) and service provision (e.g., testing), to reduce the spread of the coronavirus that causes COVID-19. However, despite the use of these behavioural strategies, cases of COVID-19 continued to persist (6), with additional emergence of variants of concern (8). Research has indicated that as the pandemic progressed in the early phases, adherence to disease prevention behaviours fluctuated across the United Kingdom population (9), fueling increasing rates of COVID-19. There have also been a number of changes to government ‘restrictions’ or prevention measures over the course of the pandemic. These may have been influenced by a number of factors, such as data, scientific modelling and political agendas. Ultimately, to reduce the continued spread of the virus, a reduction in the number of person-to-person transmissions needed to be achieved. The importance of behaviour has been clear from early on.

**Figure 1:**
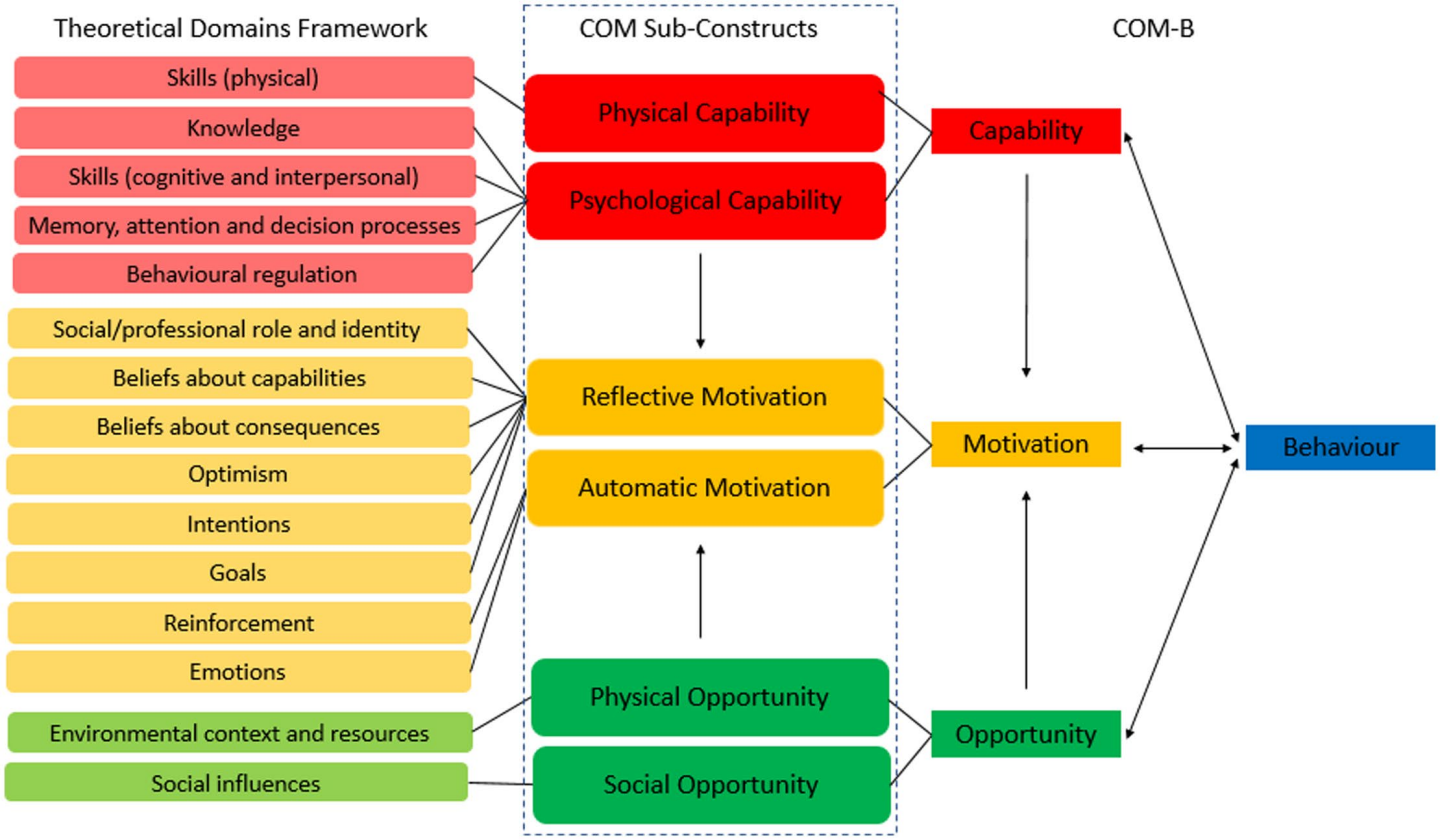
Mapping the theoretical domains framework (12) to the COM-B (4, 5) from Chater et al., (14).

Behavioural scientists have proposed principles for implementing COVID-19 specific behaviour change (10), citing theory-based interventions that are audience-appropriate and accessible, whilst ensuring that adjustments made in response to changing circumstances are clear and explained (1, 11). The COM-B model acts as a system and indicates that to perform a behaviour, an individual needs the capability, opportunity, and motivation to do so (4, 5). The Theoretical Domains Framework [TDF: (5, 12, 13)] offers a wider theoretical lens to the behavioural diagnosis using COM-B. The TDF consists of 14 domains that have been developed from the synthesis of 33 theories of behaviour (12). Figure 1 (14) highlights how the TDF and COM-B can be used together to further understand behaviour, with the original (12) domain of ‘Skills’ split into ‘Physical skills’ and ‘Cognitive and interpersonal skills’ (5, 13), to distinguish the two. Hereby, cognitive and interpersonal skills refer to the psychological ability to perform the behaviour, such as the ability to effectively communicate with others, whereas physical skills represents the physical ability to perform a behaviour.

Using this approach, behavioural diagnoses using COM-B have provided insight for COVID-19 intervention development (1, 15–17) and specifically for engagement with hand hygiene practices (18, 19), physical-distancing (20, 21), self-isolation (22) and vaccination uptake (23–26). However, it is the enactment of the constellation of behaviours together, as opposed to a single behaviour enacted in isolation, which would truly optimise COVID-19 disease prevention and public health. Traditionally, a COM-B analysis is performed on a single behaviour, ensuring that the target behaviour is specified to allow the influences on that behaviour to be identified for intervention. Understanding a constellation of COVID-19 behaviours offers a novel approach to best aid disease prevention efforts where there are limited resources and multiple, related candidate behaviours.

## 2. Aim

Three local authorities in the East of England identified schools, care homes and warehouse settings as areas of concern for increased risk of COVID-19 infection in the summer of 2021. This study aimed to investigate the core influences on COVID-19 disease prevention behaviours in these populations identified as at-risk. Brief descriptions of the identified behaviours of interest for the local authorities were: (1) hand hygiene, (2) wearing a face covering, (3) physical-distancing, (4) ventilation, (5) PCR testing when symptomatic or asked, (6) LFD testing when asymptomatic, (7) self-isolation when symptomatic or asked, and (8) vaccination uptake. The secondary aim was to produce a logic model using the Behaviour Change Wheel (4, 5), which could in turn be used to design workplace interventions.

## 3. Methods

### 3.1. Design

A qualitative design was used, with semi-structured interviews to allow for in-depth data to be generated. To ensure rigor in the study design, this study followed and is reported using the COnsolidated criteria for REporting Qualitative research (COREQ) 32-item checklist (27).

### 3.2. Participants

*Recruitment:* Participants were recruited between July–October 2021. They were eligible if they worked or were closely engaged within the three environments identified as experiencing high COVID-19 transmission (schools (S), care homes (CH), and warehouses (WH)) across three local authorities. A sign-up link (via Qualtrics), QR code and contact telephone number were circulated by community and workplace connections provided by local authorities, via posters located in community settings and sent around social media. The principal investigator (AMC) spoke on BBC Three Counties radio to promote the study and aid recruitment. In total, 260 people clicked the sign-up link. Of these, 131 did not provide any details, and 47 were not eligible due to employment setting or location (outside the local authorities of interest). One person used the telephone number, but was not eligible to participate. All of the remaining 82 eligible candidates were contacted via email, text or phone call.

*Participants:* Twenty-three participants (16 female, 7 male; 28% of those eligible) were included, selected at random until data saturation was reached. The sample was aged between 19 and 65 years old (*M* = 39.26, SD = 12.24), 14 were residents of Bedford Borough (BB), 6 of Milton Keynes (MK) and 3 of Central Bedfordshire (CB) councils; 10 worked and/or had children in schools, 8 worked in care homes, 5 worked in warehouses, and 11 lived with under 18 s. Full participant details are in Supplementary Table S1.

### 3.3. Materials

An interview schedule (Supplementary Table S2) designed by the research team made up of academics, psychologists and professionals working within a public health setting, was used to ask open-ended questions to enable participants to share their experiences during COVID-19 freely.

### 3.4. Procedure

Interviews were held between 19th July to 27th October 2021 (by PBR, a female qualitative health psychology postgraduate researcher and PhD candidate on an un-related project, with experience in qualitative research), timed to understand local behaviour following the named ‘Freedom day’ (28). At this time, in England (where the study was conducted), the national government had just relaxed a number of restrictions (e.g., no cap on number of people who can meet, 1 m-plus physical distance guidance removed, face coverings no longer required by law). To reduce the risk of COVID-19 transmission during the research, those who agreed to participate were consented and interviewed via phone call (*n* = 16), Zoom (*n* = 4) or Microsoft Teams (*n* = 3). The interviewer had no previous contact with participants and no one else was present during the interviews. An information sheet and consent form were sent prior to the interview, and consent was received prior to data collection.

Calls lasted between 33 and 64 min (*M* = 45.57, SD = 6.54). Data saturation was reached at 20 interviews and although there was an unequal number of participants across the three local authorities, the team agreed to stop at 23 when no new themes were identified (29). All conversations were recorded with the password-protected software Otter.ai and a Dictaphone to enable transcription verbatim. Transcripts were not sent to the participants following the interviews. All participants were thanked and compensated for their time with a £10 shopping voucher issued following the interview. An option to donate this voucher to their workplace was offered.

### 3.5. Ethical considerations

Ethical approval was obtained from The University of Bedfordshire Institute for Sport and Physical Activity Research (ISPAR) ethics committee, [Ref: 2021ISPAR006] and followed the British Psychological Society’s Code of Ethics and Conduct (30). Participants gave informed consent, were allocated a pseudonym and any personally identifying information was removed at transcription to maintain anonymity and confidentiality. All participants had the right to withdraw, up until 4 weeks after data collection when data was anonymised.

### 3.6. Analysis

Interview transcripts were analysed (by PBR and AMC) inductively in NVivo, using Thematic Analysis (31–33), taking an iterative approach. This type of analysis is particularly useful when conducting applied health research, as it allows researchers to conduct sophisticated and robust analysis whilst still presenting the data in an accessible format for non-academic readers (34). Transcripts were coded line by line by PBR and assigned to sub-themes. Sub-themes and overarching themes were discussed between PBR and AC and independently checked against the data. Changes to the themes were made to reduce overlap of concepts and ensure a full narrative from the data. Themes were then deductively mapped (by PBR and AC) to the TDF and COM-B and a BCW logic model was produced. This analytic approach has been used to support a needs assessment for intervention development using the BCW (35–37), including to produce recommendations for a COVID-19 vaccine uptake intervention (20). Themes were then checked with those working within a public health setting for clarity and acceptability and presented to a wider team of public health professionals to sense check meaning and understanding.

## 4. Results

**Figure 2:**
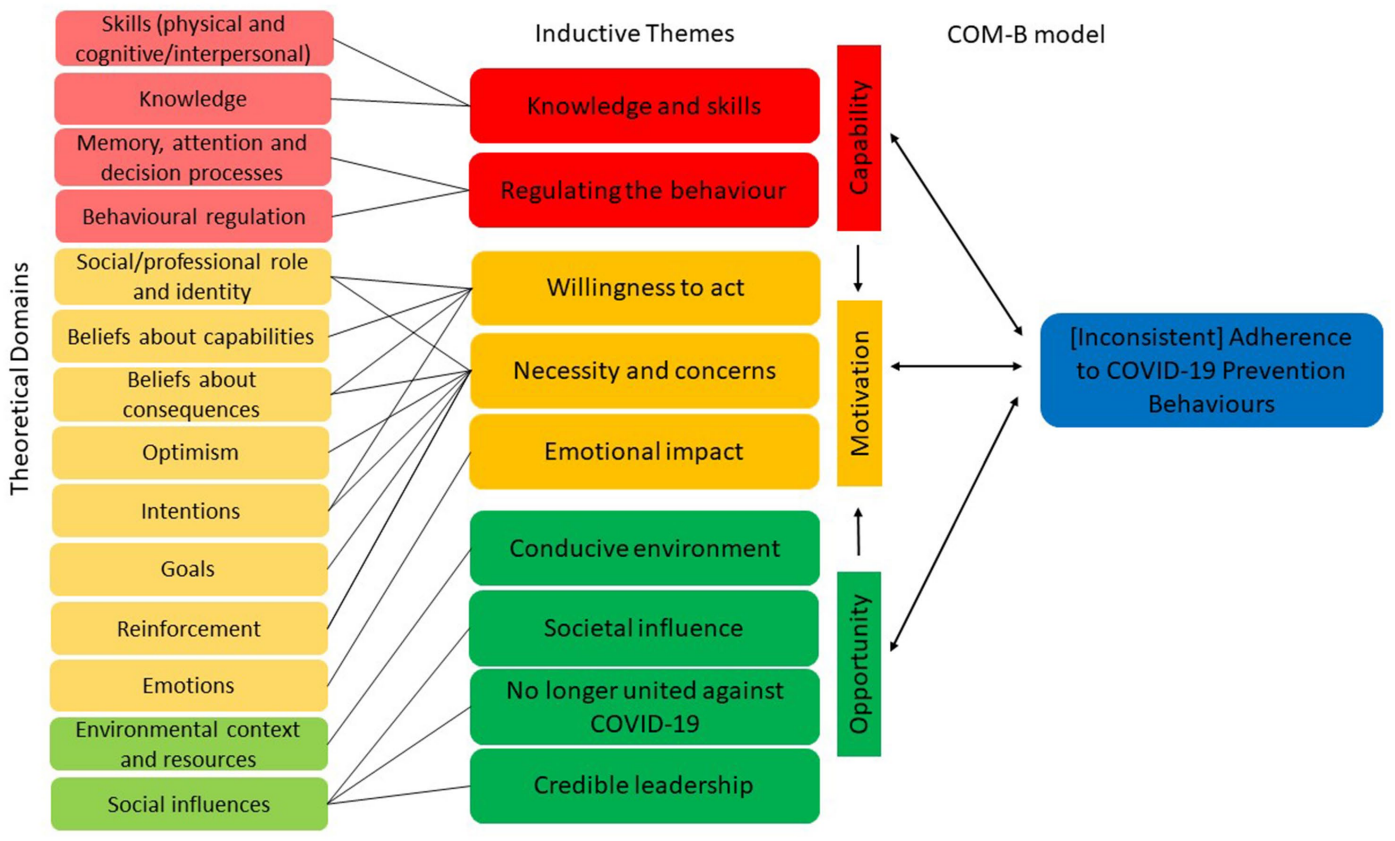
Thematic mapping of inductive themes to the theoretical domains framework and COM-B model in relation to a constellation of COVID-19 prevention behaviours.

Thematic analysis inductively identified 10 themes; (1) knowledge and skills, (2) regulating the behaviour, (3) willingness to act, (4) necessity and concerns, (5) emotional impact, (6) conducive environment, (7) societal influences, (8) no longer united against COVID-19, (9) credible leadership, and (10) inconsistent adherence to COVID-19 prevention behaviours. These themes and their mapping to the TDF and COM-B are presented in Figure 2 and in the logic model in Supplementary Table S3.

### 4.1. Theme 1: knowledge and skills

TDF = knowledge, skills; COM = psychological capability, physical capability.

#### 4.1.1. Symptoms

A multitude of symptoms for COVID-19 were given by participants. While the recognised core symptoms (a high temperature or fever, a new, continuous cough, loss or change to sense of taste or smell) were often mentioned, there were many others identified, leading participants to conclude that the core symptom messaging was diluted.

“The key symptoms are a persistent cough, a high temperature, loss of taste and smell. I know that there are others that aren't right now said by the government as key symptoms but still key to look out for.” (Annie, 31, School)

“The key ones vary so obviously the temperature, continuous cough. Lack of taste and smell, that came in afterwards, but then it's a bit more varied, it's like a runny nose, sore throat, and that funny marks on your tongue and your feet? Shortness of breath. I think it's everything nowadays.” (Tasha, 27, Care home)

“Now they’re saying it’s fatigue, sore throat, headache, and there’s six things now. So my understanding is if I lose my taste and smell. Could that be the flu like I had last year, or is it COVID? I don’t know the difference really. So then they say, aches and pains in legs as well. If you’ve got the flu, you get that as well. So I just think it’s the same as the flu.” (Sonia, 31, School)

“I think the only one that is like the common one is a cough, because everything else can be COVID right now, everything else. So I would say that if I was coughing me or my husband started to cough, and like a lot, and was feeling this very extreme fatigue right away. And then the high fever again, you know, then I would be worried.” (Aurora, 36, School)

“I think the message has been lost because, you know, so many different organisations will tell you, Oh, that's another symptom… I've got lost in what's a symptom and what's not a symptom now and I think a lot of the time they're getting confused with the common cold symptom.” (Susan, 52, Warehouse)

#### 4.1.2. Ventilation

There was confusion surrounding the knowledge of ventilation in relation to ‘Hands, Face, Space, Fresh Air’. When initially asked to describe the core COVID-19 prevention behaviours, none of the 23 participants provided ventilation as a preventive measure. When asked about the ‘Fresh air’ part of the COVID-19 messaging, some conceptualised this as needing to exercise outside whilst others questioned the benefits of opening windows.

“I think it [fresh air] actually relates to, I don't know, you can go and exercise outside?” (Laura, 36, Warehouse)

“Yeah, our windows were sort of open and ventilated, how much of a difference that made? I'm not overly sure.” (Louise, 33, School)

“I don’t think a lot of people are actually aware of the whole ventilation thing, and how it's now shown it’s more spread by airborne than on surfaces, everyone still just thinking about surfaces.” (Tasha, 27, Care home)

#### 4.1.3. Testing children

Concerns over the skills required to test young children were voiced and parents were frustrated at their inability to access appropriate guidance. Carers, however, with appropriate training and experience of testing, felt more skilled to test children. This was both in relation to the physical skills to perform the test and the interpersonal skills to put young children at ease to perform the test.

“You will never see an NHS video doing a PCR test to a child of his age, the example is always with children that know what they’re [the child] doing.” (Aurora, 36 School)

“Cause obviously I'm fully trained on doing that [PCR tests]… So cause I was able to talk to the kids and just like, because obviously I do it to myself, I was able to sort of translate like, I’m not gonna stab you with this swab. Literally it’s just gonna be a tickle.” (Lorraine, 32 Care home)

### 4.2. Theme 2: regulating behaviour

TDF = behavioural regulation, memory, attention and decision processes; COM = psychological capability.

#### 4.2.1. Not yet routine

Participants spoke of the continuous conscious effort required to adhere to the COVID-19 preventive behaviours, with frequent incidents of forgetting and a lack of the ability to regulate behaviour. For many, the behaviours had not yet become habitual, and this often led to lapses in behavioural adherence. This was commonly voiced in relation to the requirement to perform regular LFD tests, or when individuals were moving quickly between areas in the workplace which had different face covering or hand hygiene requirements. Others, however, did suggest that it had become routine.

“Sometimes I forget [to take an LFD test], but I’d say 90% of the time I’m pretty good at it.” (Suzi, 40, School)

“That tends to be when I forget to do it [wear a face covering], is when some kids need you so you instinctively rush over to help them, and I’ll forget to put the mask on.” (Michelle, 45, School)

“Hand sanitizer, again it’s quite a new thing and I think it's, you know, we’re not used to going into a shop and sanitising the hands straight away. So yeah, for me it's not the first thing I think of to do.” (Jamie, 35, Warehouse)

“I think it’s [LFD test] one of the things that you get into the habit of doing, you know like, remember to take your tablets.” (Thomas, 65, Care home)

“Well now it’s [LFD test] become a bit routine like I'm not expecting it to not being negative so if it did come back positive it'd be a surprise.” (Danielle, 39, School)

### 4.3. Theme 3: willingness to act

TDF = intentions, social/professional role and identity, beliefs about capabilities, beliefs about consequences; COM = reflective motivation.

#### 4.3.1. Intentions to adhere

Participants had the intention to adhere to the disease prevention behaviours. This included sustaining newly built routines, such as testing, or changes in behaviour when needed, such as self-isolating. Willingness was aided when participants felt capable and believed a task was easy to complete.

“I'll still stick to the same routine [for social distancing] even if it’s [LFD test] negative.” (Michelle, 45, School)

“So tonight, before I go to the hen party, I will take one [LFD test].” (Suzi, 40, School)

“I’d just follow the guidance and so I think it’s [self-isolation] 10 days max from being pinged.” (Annie, 31, School)

“I still carry on using masks in supermarkets and shopping centres.” (Juliana, 43, Warehouse)

#### 4.3.2. Part of role

The extent to which individuals felt protecting others was part of their social or professional role and identity influenced their willingness to perform the behaviours.

“I think we have an obligation to be vaccinated because we’re looking after vulnerable people.” (John, 56, Care home)

“I got them [LFD test] through work, it was a choice I didn’t have to do it but I decided to, to keep myself and everyone safe, it was the best course of action to take.” (Annie, 31, School)

“We wanted the masks to be compulsory […] we didn’t want to risk one person getting it at the time when there wasn't the vaccination available, obviously like thinking about older members of staff, those with illnesses and things like that, the same to children…” (Dean, 65, School)

### 4.4. Theme 4: necessity and concerns

TDF = social/professional role and identity, beliefs about consequences, optimism, intentions, goals, reinforcement; COM = reflective motivation; automatic motivation.

#### 4.4.1. Behaviour as protection

The necessity for the different prevention behaviours varied. The importance of performing these behaviours to protect themselves and others was commonly voiced, however, there were concerns raised over some of the behaviours, such as vaccination.

“But I feel, in order to protect ourselves and everyone else, it’s something that we need to do as a community.” (Annie, 31, School)

“The social distancing that we had at the beginning, it’s just being carried on implemented and we still have the same approach like not getting too close to a person because we were thinking the risk was high to catch the virus.” (Alex, 33, Warehouse)

“I don’t want to put my unborn child through that [vaccination].” (Ashley, 27, School)

#### 4.4.2. Route to normality

People also expressed the view that behaviours were needed as routes to achieving specific goals, such as being able to travel, see family and allow society to get ‘back to normal’.

“I’m gonna take the opportunity to be vaccinated from this virus, then hopefully the more people are vaccinated, the quicker we can get back to normal.” (Craig, 19, Care home)

“Everything comes down to, because I want to travel, to see my mom, she cannot come here.” (Juliana, 43, Warehouse)

#### 4.4.3. Wellness reinforces behavioural efficacy

Those who had not caught COVID-19, and attributed this to their adherence to the disease prevention behaviours, saw value in performing the behaviours thereby reinforcing their beliefs in necessity and adherence.

“I just think oh I've made it through another week and that’s a bonus (laughs), it’s more kind of thinking stick to it [prevention behaviours] cause obviously it's working.” (Annie, 31, School)

“I'm an example of well if you follow the protocols, then the potential for you getting it [COVID-19] is a lot less likely.” (Dean, 65, School)

This was most prevalent in workplaces where respondents spoke of the requirement of weekly testing. As participants were only taking LFD tests as a work requirement, as opposed to due to suspected COVID-19, they were expectant of a negative result. Therefore, when they received a negative result it reinforced the efficacy of their preventive behaviours and this did not change their future behaviour. Individuals stated that they would opt for a PCR test, even after a negative LFD test, if they had concerns over potential symptoms, highlighting their beliefs in the processes to prevent the transmission of the virus, to protect others.

“It’s [negative LFD test result] just a confirmation that what I’ve been doing has been working.” (Aurora, 36, School)

“I did a LFT in the morning and that came back negative. So, I was like I still don't feel like completely safe so I booked into a drive thru proper swab test.” (Michelle, 45, School)

#### 4.4.4. Waning necessity

Participants drew on their experiential knowledge of the pandemic so far to assess the necessity of engaging in the disease prevention behaviours. When participants had not been personally ill themselves, they questioned the consequences of not performing the behaviours and were optimistic about not becoming seriously ill. This seemingly influenced their intentions to perform the behaviours (e.g., wearing a face covering) in the future.

“I still haven’t had it [COVID-19]. And again if I did have it, it really obviously wasn’t that bad. So for me, having the vaccination. It kind of seems a bit redundant.” (Jamie, 35, Warehouse)

“I don’t want to go back to a face mask again. And to be honest with you I've survived long enough without it.” (Susan, 52, Warehouse)

#### 4.4.5. Concerns over side-effects

Costs for performing the behaviours were also voiced. The financial cost of testing positive and receiving reduced pay whilst isolating was raised as a concern. As was adverse reactions attributed to testing experienced by some of the participants, such as nose bleeds and tonsillitis. There were side effects to wearing face coverings experienced by some participants, such as impacting on breathing (particularly during manual labour), impaired communication, initiated panic attacks and skin blemishes, whilst visors were associated with headaches.

“They didn’t realise that employees wouldn't go there [testing tent] because they know, they will have the LFT and if it’s positive, they have to have a PCR and if it's positive, they have to isolate and they would be unpaid.” (Alex, 33, Warehouse)

“They couldn’t afford to lose the wages. At that point they had just started contracts and they wouldn't have gotten company sick pay…” (Jamie, 35, Warehouse)

“I mean every time I touch my tonsils, I get tonsillitis…” (Lorraine, 32, Care home)

“The mask, has taken a toll on my skin after a while…” (Michelle, 45, School)

### 4.5. Theme 5: emotional impact

TDF = emotions; COM = automatic motivation.

#### 4.5.1. Feeling the impact

There was an emotional toll from the pandemic that most frequently took the form of fear, sadness, frustration, anger, and relief. The disease prevention behaviours were a reminder to participants of the impact the pandemic has had on their lives.

“The government has put way too much effort on this coronavirus thing and it has to stop, it has to stop because it takes too much mentally.” (Alex, 33, Warehouse)

“That was very, very hard on our family, and it broke us, a little bit it broke us, not joking, it broke us a little bit, all this nonsense of, you know, testing or not testing, coming home or not coming home. You’re always stressed, like your adrenaline levels are always this high.” (Aurora, 36, School)

#### 4.5.2. Emotional responses

Many of the participants’ frustrations were exacerbated as they felt they were putting themselves and their families at risk by going to work. At times, as emotions became overwhelming, this impacted on adherence to the preventive behaviours.

“To be honest with you walking in and seeing all the empty shelves [in supermarkets] it’s just soul destroying after a day at work. You know, and having to follow a one way system … I mean, to the point, this woman had a go at me because I'd walked the wrong way down an aisle and I went, 'how many hours have you worked today?!’” (Susan, 52, Warehouse)

“I don't like doing that [testing] at all. My anxiety goes through the roof every time I have to do a test, because I know how long I suffer after the test.” (Lorraine, 32, Care home)

#### 4.5.3. Turmoil of testing young children

Parents were left exasperated by testing their young children, describing the experience as ‘terrifying’ and a ‘nightmare’.

“With a two-year old, pining them down, trying to shove it up their nose and it's, yeah, it's horrible.” (Lueanna, 32, School)

“We spend like often half an hour, 40 minutes trying to grab him, two adults, we're not able to safely grab him, because the thing is like you cannot grab your child to a point because you know when you put that on the nose. If it does like this, it can hurt his nose. So, so yeah. After that morning when I nearly cried, because at that point it was a third one, I said this is ridiculous, because only people that have never tried to do this on a child at this age would make you do one.” (Aurora, 36, School)

### 4.6. Theme 6: conducive environment

TDF = environmental context and resources; COM = physical opportunity.

#### 4.6.1. Challenging environments

Some work environments made the disease prevention behaviours more difficult. They did not allow for physical-distancing (i.e., when providing personal care in care homes) or would necessitate major structural changes to increase handwashing (i.e., relocating sinks).

“It has been hard, I mean, when we do personal care, we have to get within close proximity.” (Lorraine, 32, Care home)

#### 4.6.2. Adapted environments

Adaptations made to the working environment, however, were raised as contributing factors to disease prevention behavioural adherence. Workplaces had provided individuals with the physical opportunity to perform the behaviours, by providing face coverings, tests, hand sanitiser, modifying seating arrangements and staggering shift patterns. Furthermore, some workplaces had scheduled testing time into the working day and others provided transport to vaccination centres.

“They provide a lot of things, I mean, everybody can find hand sanitizer. I mean, they work a lot too, to keep the place as safe as possible, even in the canteen, they reduce the chairs at the table.” (Laura, 36, Warehouse)

“We've all been allocated [time to test] on our timetables.” (Tasha, 27, Care home)

#### 4.6.3. Structural support

Behaviours were also supported by effective systems. Participants spoke highly of the processes for booking PCR testing, enabling them to easily access tests and receive timely results.

“Very easy, very straightforward. Local one was a five-minute drive away. Very clear instructions on what to do when you arrive, what to expect. How long results will take, they were actually much faster at that point which was really good as well.” (Ashley, 27, School)

### 4.7. Theme 7: societal influence

TDF = social influences; COM = social opportunity.

#### 4.7.1. Conforming to the norm

The perception of judgement from others was a contributing factor to enacting a behaviour. Participants referenced adjusting their adherence to behaviours based on the actions of others, this was particularly prevalent for wearing a face covering.

“You kind of think ‘Should I wear a mask?’, ‘Are a lot of people in masks?’ You know, just to save, I don't know, maybe people looking at you funny.” (Jamie, 35, Warehouse)

“You have to do what everyone else is doing, otherwise you look the odd one out don't you. Like wearing a mask at work, I hate wearing a mask at work, especially when it's hot and everything else. And, you know you're running around and you're out of breath, but you can't take your mask off, because everyone else at work is wearing their masks.” (Sonia, 31, School)

#### 4.7.2. Feeling pressured

The social implications of disrupting the lives of others by spreading COVID-19 was also factored into people’s decisions to test themselves and their children and to maintain physical-distancing structures. Societal pressure was heightened in workplaces which chose to overtly monitor the behaviour of their employees.

“I'm usually good [at testing] on Sunday because I sort of, I just feel I don't want to send them in [to school] and then find out that, actually, we've got it […] Some people send their children in with symptoms and their bubbles have to be closed. So it's more of a societal pressure there, from that perspective.” (Danielle, 39, School)

“I feel like I'm harassed by the social-distancing auditors because they are looking after you or chasing you, making sure that you don't step on the other side, even now.” (Alex, 33, Warehouse)

#### 4.7.3. Feeling empowered

Individuals reported feeling supported within their social environments to perform preventive behaviours. In particular, in workplaces where senior leadership teams worked closely with their staff, individuals felt empowered to speak up if they had any safety concerns.

“We were told to and advised to and also very much given, SLT [senior leadership team] gave us the power as such, to at any point if we were uncomfortable, to voice that you're uncomfortable and that it's okay to say 'you're not wearing a mask, can we not', or 'you're too close, please back away', so that was quite nice and that message was constantly reminded to us, you know, please say if you're uncomfortable.” (Louise, 33, School)

#### 4.7.4. Supportive teams

Equally workplaces which successfully delivered the message that they valued their staffs’ safety, had staff who perceived less barriers to enacting the behaviours.

“Basically it's just you know, ‘Protect yourself. Protect your family, if you need any support. If you are going for PCR test, don't worry about work. We'll be here.’ They're very supportive.” (Sonia, 31, School)

### 4.8. Theme 8: no longer united against COVID-19

TDF = social influences; COM = social opportunity.

A societal disconnect was frequently discussed, conveying that the United Kingdom was no longer united against COVID-19. This manifested itself in two ways; mistrust of the media and frustration at others.

#### 4.8.1. Mistrust of the media

Participants spoke of an awareness that ‘the news’ was not to be taken at face value and opted to either fact check, or to completely avoid mainstream media. Respondents had a sense that news outlets had their own agendas, leaving them without a clear trusted knowledge source.

“I do think the news obviously can be a bit biased so I will listen to their news stories and what they're saying and tend to look more at links they've credited, where they've got their research from, and go to them.” (Michelle, 45, School)

#### 4.8.2. Frustration with others

Throughout the COVID-19 pandemic people’s personal experiences have varied widely, as have their capability, opportunity, and motivation to perform the disease prevention behaviours. However, participants expressed frustration that others did not behave in line with their expectations. The divide between people seemed to have grown over time, and left people feeling disunited and often frustrated with those in power.

“I think people are now on two sides, people that believe that we should still be locked down and we should still be having all these restrictions and then the other people on my side of the fence who want to just crack on now.” (Lueanna, 32, School)

“It does frustrate me because I've had, I've witnessed my [care home] residents, have witnessed people die from that, so there's all of this stuff. And yet you're [others] perfectly fine, not testing, spreading the disease.” (Lorraine, 32, Care home)

“We are not on the same boat anymore because everyone is doing their own thing.” (Aurora, 36, School)

“I don't understand why they've [the government] just said, ‘Oh no, we don't need it, it becomes this, your choice,’ if you give people choices, they will, too many people will, choose the wrong option.” (John, 56, Care home)

“And then other people are misinformed or don't quite understand, and then therefore, it just gets to the point where you think well why am I following the guidance when other people aren't doing it.” (Annie, 31, School)

### 4.9. Theme 9: credible leadership

TDF = social influences; COM = social opportunity.

#### 4.9.1. Practice what they preach

Adherence to COVID-19 disease prevention behaviours was also influenced by access to credible leadership. Instances in which politicians had not adhered to the prevention behaviours were cited directly as reasons to question the purpose and benefit of behavioural adherence. The frequency with which political missteps have been made public was raised as causing a reduction in people’s willingness to do what they were advised.

“It’s the same as the G7 summit, when they were all hugging at the barbecue. So we see politicians doing it, so when I have social time with my friends and family why can't I be doing the same sort of thing?” (Annie, 31, School)

“I think by the time we had Mr. Cummings in the back garden of Number 10 And then Matt Hancock. I think a lot of people, they gave up and you kind of think that's it, it's [the country] leaderless.” (Thomas, 65, Care home)

#### 4.9.2. Who is being protected?

Similarly, when senior leaders in the workplace were seen to be flouting the rules or not following disease prevention behaviours, it provided an instant rebuttal and devalued their request for others to adhere to any workplace guidance. This left employees to doubt the value of new guidance, thereby minimising behavioural buy-in.

“I seen her [member of Senior Leadership Team] walking around without her face mask on so it's kind of like, you know, you can't tell us all to wear our face masks when you're going to walk around the warehouse not wearing a face mask.” (Jamie, 35, Warehouse)

“I think it [mask wearing at work] was more done to shut people up than it was anything else. You know, rather than have any sort of like good basis to help stop the spread.” (Susan, 52, Warehouse)

### 4.10. Theme 10: inconsistent adherence to COVID-19 prevention behaviours

COM-B = behaviour.

The variation in people’s capability, opportunity, and motivation resulted in varying adherence to the COVID-19 prevention behaviours. This was evidenced through variability in performance (or not) of each behaviour addressed in this study.

#### 4.10.1. Hand hygiene (hands)

“Before they go to lunch, when they're coming in from break, we get them to just gel their hands.” (Dean, 65, School)

“I mean none of them [colleagues] are washing their hands, none are using the sanitising stations.” (Jamie, 35, Warehouse)

#### 4.10.2. Face coverings (face)

“I wear a face mask at work.” (Jamie, 35, Warehouse)

“I don't wear one [face covering].” (Susan, 52, Warehouse)

#### 4.10.3. Physical-distancing (space)

“We avoid touching each other, hugging or stuff like that.” (Laura, 36, Warehouse)

“[Colleagues] are not keeping the distance, hugging each other, even during the last year. During the peak epidemic, it was really bad at my work.” (Jamie, 35, Warehouse)

#### 4.10.4. Ventilation (fresh air)

“Our windows were sort of open and ventilated.” (Louise, 33, School)

“They're only keeping the doors open when they're trying to load lorries.” (Susan, 52, Warehouse)

#### 4.10.5. Testing (find, test)

“I have to do it [LFD test], I think it's a Wednesday evening or Thursday morning for work and I have to log it on the government website.” (Sonia, 31, School)

“The doctor said, ‘No no no, you should get a PCR test' and I said 'No, I'm not going to get a PCR test.” (Susan, 52, Warehouse)

#### 4.10.6. Self-isolation (isolate; and using the app to be identified as a contact for isolation)

“Yeah, the minute I found out [identified as a contact of someone who tested positive], I contacted my school and went into isolation for 10 days after.” (Michelle, 45, School)

“I’ve got a four-year-old, who is joined to my hip, and he wanted to cuddle, he wanted to kiss, but [when isolating] as soon as he walked into my room, he had to have a mask on, he had to have a face shield and he had white gloves.” (Sonia, 31, School)

“Yeah, I’ve got it on my phone [track and trace app]. When I go to places like restaurants, I use the check in.” (Annie, 31, School)

“We haven’t got the app [track and trace], I don’t want the app. We all deleted the app.” (Sonia, 31, School)

#### 4.10.7. Vaccination

“I've been double jabbed.” (Tasha, 27, Care home)

“It's [the vaccine] not something I'm prepared to have myself.” (Ashley, 27, School)

## 5. Discussion

Behaviour is central to COVID-19 disease prevention (1, 11). This research aimed to understand what influences a constellation of eight disease prevention behaviours: (1) hand hygiene; (2) wearing a face covering; (3) physical-distancing; (4) ventilation; (5) PCR testing when symptomatic or asked; (6) LFD testing when asymptomatic; (7) self-isolation when symptomatic or asked; and (8) vaccination uptake. Using inductive thematic analysis ten themes were identified; (1) knowledge and skills; (2) regulating the behaviour; (3) willingness to act; (4) necessity and concerns; (5) emotional impact; (6) conducive environment; (7) societal influence; (8) no longer united against COVID-19; (9) credible leadership; and (10) inconsistent adherence to COVID-19 prevention behaviours.

A deductive COM-B behavioural diagnosis was performed, mapping themes to capability, opportunity, and motivation, along with their relevant theoretical domains from the TDF. All six of the COM-B constructs were identified in the themes, with psychological capability, reflective motivation and social opportunity the most commonly represented. All TDF domains were identified as relevant, namely: (1) knowledge, (2) skills (physical and cognitive/interpersonal), (3) social/professional role and identity, (4) belief about capabilities, (5) optimism, (6) belief about consequences, (7) reinforcement, (8) intentions, (9) goals, (10) memory, attention and decision processes, (11) environmental context and resources, (12) social influences, (13) emotions, and (14) behavioural regulation.

### 5.1. Capability

There were clear gaps in psychological capability which contributed to behavioural non-adherence. Knowledge gaps were particularly prevalent regarding symptoms and ventilation, with confusion over the core and additional symptoms of COVID-19 and a lack of awareness of the importance of ventilation. Other research has shown that 40.4% United Kingdom participants were unable to identify the three primary COVID-19 symptoms (38). The Behaviour Change Wheel (4, 5) suggests knowledge can be improved via education which may be provided through communications or marketing. It has been highlighted that health messaging works best when designed in collaboration with the target audience (17), therefore local authorities and employers should aim to co-create their communication and marketing strategies with their local residents and staff.

Where participants understood COVID-19 disease prevention behaviours, there were still instances of difficulties in behavioural regulation. This mirrors a COM-B analysis of hand hygiene in the early stages of the COVID-19 pandemic, which found psychological capability, social opportunity, and reflective motivation to influence this behaviour (19). Behaviour change techniques such as goal setting, action planning and feedback on behaviour could support individuals to better regulate their behaviour. Implementation intentions are a form of action planning, shown to be an effective way for individuals to set goals and create plans to achieve them (39, 40). Communication and marketing, alongside service provision, could enable individuals to make implementation intentions, encouraging the consideration of a situation (If) and an action (Then) to identify ways to regulate behaviour and develop new habits. For example, ‘If I am about to enter the door of the supermarket, then I will clean my hands with my hand gel before touching the door’ (18). As memory was also highlighted as an issue for behavioural adherence, prompts and cues, for example signage could overcome this.

There were also gaps in capability to perform behaviours. In particular, participants felt they did not possess the interpersonal and physical skills to test young children, both at home and at mobile PCR units. However, those trained to perform tests felt they had the skills to perform this behaviour, suggesting a roll out of wider training could address this issue. Skills can be enhanced through Behaviour Change Techniques (41) such as 4.1 ‘instruction on how to perform the behaviour’, 6.1 ‘demonstration of the behaviour’, and 8.1 ‘behavioural practice/rehearsal’, which could all be delivered via the intervention strategies (4) of training and modelling. As such, local authorities and employers may benefit from working with community members and staff when designing communication, marketing, and training to increase public capability to perform these behaviours.

### 5.2. Opportunity

There was variation in opportunity to perform behaviours in both a physical and social context. The degree to which working environments had been, or were able to be, adapted had an impact on behavioural adherence. This aligns with previous COM-B analysis of physical-distancing and hand hygiene, which has shown physical opportunity can act as a barrier or enabler to these behaviours (19, 20). Restructuring the physical environment where feasible, and adding objects into the environment at work, can change a barrier to an enabling factor. Governments could go further in offering wider financial support to cover sickness to enable staff to stay off work when testing positive for COVID-19 and access to tests, fresh air, hand sanitiser, face coverings and space to enable distancing.

The social environment was also found to influence behavioural adherence, with participants citing the influence of others as a factor for behaviours such as wearing a face covering. This aligns with studies that have shown wearing a face covering to be the behaviour most adhered to, with the suggestion this may be due to social pressures to conform (42, 43). Using modelling to show others wearing face coverings as a demonstration of the behaviour, alongside encouraging social comparison and providing information about other’s approval, may therefore increase the likelihood of behavioural adherence, through increasing social influences via social opportunity.

In contrast, the current study also found evidence for a societal divide, resulting in a sense of no longer being united against COVID-19. This is in line with a longitudinal United Kingdom study which found the emergence of two partisan groups, merged across pre-existing societal divides such as income and/or education level, that were split by behavioural adherence (44). Trust, or indeed distrust of health officials and science has been cited as the dividing factor of these groups. It is, therefore, important, to highlight commonalities within their communities, using unifying messages, and the concept of embracing the ‘we’ and minimising the ‘I’ (1) to conquer division. This approach has been recommended by a recent systematic review (17) and guidance (16) produced by the British Psychological Society’s (BPS) COVID-19 Behavioural Science and Disease Prevention (BSDP) Taskforce.

Ensuring trust in public health messaging and the messengers behind the message is important, with research highlighting the need for Transparency, cRedibility, Unified messaging, Social responsibility and norms, and Timely messaging [TRUST: (16)]. A focus on retaining trust must also involve providing access to credible leaders. Participants in this research questioned credible leadership at both an organisational and governmental level, with a call for leaders to practice what they preach. With regards to the government, this aligns with the sudden drop in public trust that was recorded after the defence of Dominic Cummings’s (a high profile Chief Advisor to the United Kingdom government) breach of COVID-19 guidance (9). This supports other research that has found that seeing authority figures acting against protection measures and the ‘rules’ can be a reason to not adhere to disease prevention behaviours such as physical-distancing (20). There is a need for credible leaders to demonstrate adherence to disease prevention behaviours, being positive social influences on behaviour.

### 5.3. Motivation

Conscious thought processes also contributed to behavioural adherence through a willingness to act and beliefs related to necessity of action and perceived concerns of consequences. These influenced people’s reflective motivation, via a pathway of costs and benefits, which can be further explained via the Necessity-Concerns Framework [NCF: (45)], which posits that adherence, often researched in the field of medication or treatment optimisation, is dependent on the perception of the need, and concerns about unintended outcomes or side effects. When participants did not perceive high need for a disease prevention behaviour, it was less likely to be enacted. This was often reinforced by lack of infection, and can be related to beliefs about consequences and perceived risk. A recent study of vaccine intentions in keyworkers reported that vaccine hesitancy was associated with a low perceived risk of becoming infected within 6 months (46), which may impact necessity beliefs. Although it may be intuitive to attempt to increase perceptions of risk, vaccination campaigns which over-emphasise the dangers of viruses can create negative beliefs (25). In contrast, research has found that information on benefits can be effective (47) for those who were vaccine-hesitant, and that a focus on personal benefit as opposed to collective is most beneficial. This compliments recommendations to tailor messages for targeted groups by engaging communities in the design of communication campaigns (17). Therefore, future public health messaging should engage communities to understand their specific goals (such as seeing family, travel, and getting back to normality) and beliefs about consequences (e.g., lower infection rates, fewer lockdowns), rather than fears, to produce the most effective health messaging. Behaviours were also more likely to occur when people felt it was part of their role or identity, such as carers and teachers. Being optimistic about the outcome, and having a positive belief in the ability to perform the behaviour, such as when it felt ‘easy’ were also influential factors to behavioural performance. These are important aspects of the EAST framework from the Behavioural Insights Team (48), which suggests that interventions should be Easy, Attractive, Social and Timely.

The NCF equally postulates that a reduction in behavioural adherence can occur due to concern over side effects (45). In the current study disease prevention behaviours were related to concern for a number of costs, such as bad skin following wearing a face covering, or financial loss from lack of pay when self-isolating. These costs have similarly been noted in a recent systematic review (49). Controlled, outdoor, and scheduled breaks from wearing a face covering for example, may be an effective technique for workers who are required to wear them throughout their working day in an attempt to reduce the perceived cost.

Behaviours may also be avoided due to unconscious drives, linked to emotion or habit, representing automatic motivation. This study highlights that COVID-19 and the related disease prevention behaviours have taken an emotional toll. People have felt isolated, which has been exacerbated during periods of lockdowns and self-isolation, and behaviours such as testing have caused feelings of anxiety. The Office for National Statistics (50) show an increase in levels of depression in the United Kingdom since pre-pandemic figures (10% pre-pandemic; 17% summer 2021), now experienced by 1 in 6 adults. This could lead to lapses in physical-distancing, which may be linked to unconscious or automatic interference with planned COVID-19 disease prevention behaviours, driven by emotion and habit when seeking emotional and mental health support from others (20). Low mood, isolation and loneliness may make other behaviours more difficult, such as booking and going for a COVID-19 test. Furthermore, presenting public health messages as contrary to social norms can increase feelings of uncertainty, leading to maladaptive behaviours, such as panic buying, to alleviate such concern (51). Lack of public worry, on the other hand, has been linked in previous pandemics, to low performance of disease prevention behaviours such as hand hygiene and physical distancing (52). As such, it is important to attend to emotional state whilst trying to increase COVID-19 disease prevention behavioural adherence so that messaging provides a basis for motivating appropriate action rather than triggering unintended or detrimental feelings and actions. This echoes the Psychological Guidance provided by the BPS COVID-19 Behavioural Science and Disease Prevention Taskforce, who recommend creating worry but not fear (1).

### 5.4. Limitations

The investigation of a constellation of behaviours could be considered as a limitation of this study given that the BCW (4, 5) guides users to specify one target behaviour for a COM-B analysis. However, this approach was chosen to offer detailed insight that could be used by the commissioning local authority public health teams. While investigating behaviours in isolation can offer specificity, the current approach provides a novel way of thematically capturing shared influences across COVID-19 disease prevention behaviours using qualitative methodology. This could therefore be seen as a strength to the study as this can enable teams to spend limited budgets with greater insight across multiple behaviours. Although this may have missed more complex relationships where the factors influencing one prevention behaviour impacted on other related behaviours (53). Future research should look at the benefits and restrictions of performing a COM-B analysis on a selection of related behaviours as opposed to a single target behaviour.

Despite efforts to engage, the study was limited by the low representation of people from ethnic minority backgrounds, who have a high residency in the areas sampled and are commonly under-reached. This is in spite of efforts made by the leads for the school, care home and warehouse workplaces to recruit a diverse population. Anecdotal feedback highlighted concern with participation in the study, and speaking out of turn about their workplace. This, in addition to the small sample size traditionally used in qualitative research, limits the generalisability of the results of this study. Future work would benefit from facilitating community champions and widening routes to access to ensure all representative voices are heard, reinforcing the assurances of anonymity.

### 5.5. Conclusion

This study provides an novel COM-B and TDF analysis of a constellation of eight COVID-19 prevention behaviours in high risk-of-infection work settings in three local authorities. Through thematic mapping and production of a logic model, the study has provided direction for future intervention. Priority should be given to target individuals’ psychological and physical capability, physical and social opportunity, reflective and automatic motivation to maximise behavioural adherence. Organisations who are adapting their work environment to enable COVID-19 disease prevention behaviours should be commended. Leaders should ensure that they are a credible source and model appropriate and non-conflicting behaviours. Clear and transparent information and demonstration on how to perform behaviours should be made freely available and accessible, and strategies to prompt memory and behavioural regulation should be used. When changes to guidance are necessary, it should be made clear what the new guidance is, specifically related to behavioural actions and their efficacy and/or consequences. Messages should be unified and promote social responsibility and positive social influences.

Findings from this study offer an extended approach to the understanding of a constellation of COVID-19 disease prevention behaviours, offering a combined COM-B diagnosis for related behaviours. Findings from this research support the Behavioural science and disease prevention: Psychological guidance produced by the BPS COVID-19 Behavioural Science and Disease Prevention Taskforce (1, 15), which emphasizes: (1) the need for a collective viewpoint where society focuses on the ‘we’ rather than the ‘I’, (2) to identify clear behavioural action and influences on behaviour taking into account capability, opportunity and motivation, (3) to consider emotional reactions to public health policies and campaigns, (4) for interventions to use credible sources when providing information and modelling behaviour, and (5) to ensure channels for health information are accessible and avoid creating inequity. Collectively, these issues have organically been identified from the current data set, highlighting the value of behavioural science and a psychological evidence-based response. This further strengthens the call (1, 3) for the use of and investment in behavioural science and health psychology to optimise health and wellbeing.

## Data availability statement

The data supporting the conclusions of this article will be made available by the authors, without undue reservation.

## Ethics statement

The study was reviewed and approved by ISPAR Ethics - University of Bedfordshire. The participants provided their written informed consent to participate in this study.

## Author contributions

AMC, PB-R, FT, EC, JH, LBMTB-D, AM, GWS, TE, AK, DBO’C, EW, LJML, EM, VS, and MAA contributed to the conception of the study, commissioned by FT and EC representing three local authorities and informed by the work of the British Psychological Society COVID-19 Behavioural Science and Disease Prevention Taskforce for which AMC was Chair and JH, LBMTB-D, GWS, TE, AK, DBO’C, EW, LJML, EM, VS, and MAA were all members. AMC, PB-R, and FT developed the study design. FT and EC facilitated recruitment. PB-R conducted the interviews. PB-R and AMC analysed the data and wrote the first draft of the manuscript. All authors contributed to the article and approved the submitted version.

## Funding

This research was funded by Bedford Borough, Central Bedfordshire and Milton Keynes Councils, internally led by FT and EC, awarded to AMC at the ISPAR Centre for Health, Wellbeing and Behaviour Change, University of Bedfordshire. It was informed by the work of the British Psychological Society COVID-19 Behavioural Science and Disease Prevention Taskforce, which partially funded AMC time.

## Conflict of interest

The authors declare that the research was conducted in the absence of any commercial or financial relationships that could be construed as a potential conflict of interest.

## Publisher’s note

All claims expressed in this article are solely those of the authors and do not necessarily represent those of their affiliated organizations, or those of the publisher, the editors and the reviewers. Any product that may be evaluated in this article, or claim that may be made by its manufacturer, is not guaranteed or endorsed by the publisher.
