# Supplementary material for: Understanding a constellation of eight COVID-19 disease prevention behaviours using the COM-B model and the theoretical domains framework: a qualitative study using the behaviour change wheel
Source: Front Public Health. 2023 Jul 5;11:1130875. doi: 10.3389/fpubh.2023.1130875 (PMC10355219; doi:10.3389/fpubh.2023.1130875)
Supplement: Supplementary file 1 [file Table_1.docx]

**Supplementary Table 1: Participant demographics, location, work setting and children at home**

| **Pseudonym** | **Age** | **Gender** | **Ethnicity** | ***Ethnicity** | **Local Authority** | **Setting** | **Children at home** |  |
| --- | --- | --- | --- | --- | --- | --- | --- | --- |
|  |  |  |  |  |  |  |  |  |
| Craig | 19 | Male | White | British | BB | CH | No |  |
| Lorraine | 32 | Female | White | British | BB | CH | Yes |  |
| John | 56 | Male | White | British | BB | CH | No |  |
| Lucy | 58 | Female | White | British | BB | CH | No |  |
| Tasha | 27 | Female | White | British | BB | CH | No |  |
| Annie | 31 | Female | White | British | BB | S | No |  |
| Danielle | 39 | Female | White | British | BB | S | Yes |  |
| Aurora | 36 | Female | White | Other white | BB | S | Yes |  |
| Ashley | 27 | Female | Mixed | White & Black Caribbean | BB | S | Yes |  |
| Louise | 33 | Female | White | British | BB | S | No |  |
| Sonia | 31 | Female | White | British | BB | S | No |  |
| Michelle | 45 | Female | White | British | BB | S | Yes |  |
| Dean | 65 | Male | White | British | BB | S | No |  |
| Suzi | 40 | Female | White | British | BB | S | Yes |  |
| Jamie | 35 | Male | Other | Arab | MK | WH | Yes |  |
| Alex | 33 | Male | White | Other white | MK | WH | No |  |
| Laura | 36 | Female | White | Other white | MK | WH | No |  |
| Juliana | 43 | Female | White | Other white | MK | WH | Yes |  |
| Thomas | 65 | Male | White | British | MK | CH | No |  |
| Vali | 35 | Male | White | Other white | MK | CH | Yes |  |
| Marie | 33 | Female | White | Other white | CB | CH | Yes |  |
| Susan | 52 | Female | White | British | CB | WH | No |  |
| Lueanna | 32 | Female | White | British | CB | S | Yes |  |

*British includes English, Welsh, Scottish, Northern Irish or British

BB = Bedford Borough, MK = Milton Keynes, CB = Central Bedfordshire

CH = Care homes, S = Schools, WH = Warehouses
