# Supplementary material for: Understanding a constellation of eight COVID-19 disease prevention behaviours using the COM-B model and the theoretical domains framework: a qualitative study using the behaviour change wheel
Source: Front Public Health. 2023 Jul 5;11:1130875. doi: 10.3389/fpubh.2023.1130875 (PMC10355219; doi:10.3389/fpubh.2023.1130875)
Supplement: Supplementary file 2 [file Table_2.docx]

**Supplementary Table 2: Interview schedule to investigate the constellation of COVID-19 disease prevention behaviours**

| **Questions** |
| --- |
| 1. What would you say are the key behaviours to reduce the spread of COVID-19? |
| 1. *Script to read: The government has asked the public to clean their hands, socially distance, wear face coverings and ensure good ventilation to reduce the likelihood of catching or spreading COVID-19 using the phrase hands-face-space and fresh air.* 2. What is your understanding of these behaviours? |
| 1. How do you think these behaviours will help to reduce the risk of catching or spreading COVID-19? |
| 1. Thinking about these behaviours, what is your experience of doing these in your day-to-day life? |
| 1. What situations make it less likely for you to perform these behaviours? |
| 1. What might make these behaviours challenging? |
| 1. What would need to change to make them easier? |
| 1. What is your understanding of the key symptoms of COVID-19? |
| 1. Have you ever had any of these symptoms? |
| 1. What do you know about COVID-19 testing? |
| 1. Have you ever had a test for COVID-19? |
| 1. What was the experience like for you? |
| 1. Between having your test and getting your result, did you self-isolate? *Tell me about this time.* |
| 1. What was the result of your COVID-19 test? |
| 1. Did the behaviours we were talking about earlier change at all following your negative test result? |
| 1. Are you aware people can now get a lateral flow test for COVID-19 even if they don’t have symptoms? |
| 1. Have you ever had one? |
| 1. What would stop you from having one of these tests? |
| 1. Would having a negative test result from one of these tests change whether you continued with the behaviours we discussed earlier (hand-cleaning/ social distancing/ wearing a face covering)? |
| 1. Have you ever been asked to self-isolate during the pandemic? |
| 1. What would happen if you were asked to self-isolate tomorrow? |
| 1. Have you ever heard of the NHS track and trace app? |
| 1. What are you views around the COVID-19 vaccination? |
| 1. What do you think influences people’s decision around getting the vaccine? |
| 1. How you feel about the recent [July 19^th^ 2021] changes? |
