# Supplementary material for: Understanding a constellation of eight COVID-19 disease prevention behaviours using the COM-B model and the theoretical domains framework: a qualitative study using the behaviour change wheel
Source: Front Public Health. 2023 Jul 5;11:1130875. doi: 10.3389/fpubh.2023.1130875 (PMC10355219; doi:10.3389/fpubh.2023.1130875)
Supplement: Supplementary file 3 [file Table_3.docx]

**Supplementary Table 3. Logic model of thematic analysis mapped to the Behaviour Change Wheel in relation to the COM-B model, Theoretical Domains Framework, intervention strategies (intervention types and policy options) and behaviour change techniques (BCTs) for COVID-19 disease prevention behaviours**

| **COM-B construct** | **TDF domain** | **Theme** | **Intervention type** | **Policy option** | **Behaviour Change Technique (BCT) with codes for the BCT Taxonomy version 1 (Michie et al., 2013)** |
| --- | --- | --- | --- | --- | --- |
| Capability  (Psychological) | Knowledge | *Knowledge and skills* | Education | Communication  /Marketing | 4.1 Instruction on how to perform the behaviour  5.1 Information about health consequences  2.2 Feedback on the behaviour  2.3 Self-monitoring of behaviour  2.7 Feedback on outcome(s) of behaviour |
|  | Skills (Cognitive and interpersonal) | *Knowledge and skills* | Education  Training  Modelling | Communication/ Marketing  Guidelines | 2.2 Feedback on the behaviour  2.3 Self-monitoring of behaviour  4.1 Instruction on how to perform the behaviour  5.1 Information about health consequences  5.6 Information about emotional consequences  6.1 Demonstration of the behaviour  8.1 Behavioural practice/rehearsal |
|  | Memory, attention and decision processes | *Regulating the behaviour* | Enablement  Training | Environmental/ Social planning  Service provision | 1.1 Goal setting (behaviour)  1.2 Problem solving  1.4 Action planning  1.5 Review behaviour goal(s)  7.1 Prompts/cues  12.1 Restructuring of the physical environment  12.5 Adding objects to the environment |
|  | Behavioural regulation | *Regulating the behaviour* | Education  Training  Enablement | Communication/ Marketing  Environmental/ Social planning  Service provision  Guidelines | 1.1 Goal setting (behaviour)  1.2 Problem solving  1.4 Action planning  1.8 Behaviour contract  1.9 Commitment  2.2 Feedback on behaviour  2.3 Self-monitoring of behaviour |
| Capability  (Physical) | Skills | *Knowledge and skills* | Education  Training  Modelling | Communication/ Marketing  Guidelines | 2.2 Feedback on the behaviour  4.1 Instruction on how to perform the behaviour  6.1 Demonstration of the behaviour  8.1 Behavioural practice/rehearsal |
| Opportunity  (Physical) | Environmental context and resources | *Conducive environment* | Environmental restructuring  Restriction  Enablement | Environmental/ Social planning  Guidelines  Regulation  Legislation  Service provision  Fiscal measures | 7.1 Prompts/cues  12.1 Restructuring the physical environment  12.2 Restructuring the social environment  12.5 Adding objects to the environment |
| Opportunity  (Social) | Social influences | *Societal influence*  *No longer united against COVID-19*  *Credible leadership* | Modelling  Enablement | Communication/ Marketing  Environmental/ Social planning | 2.1 Monitoring of behaviour by others without feedback  3.1 Social support (unspecified)  3.2 Social support (practical)  3.3 Social support (emotional)  5.3 Information about social/environmental consequences  6.1 Demonstration of the behaviour  6.2 Social comparison  6.3 Information about others’ approval  9.1 Credible source  10.4 Social reward |
| Motivation  (Reflective) | Social/  professional role and identity | *Willingness to act* | Education  Persuasion  Modelling | Communication/ Marketing  Environmental/ Social planning | 13.1 Identification of self as role model  13.3 Incompatible beliefs  13.4 Valued self-identity  13.5 Identity associated with changed behaviour |
|  | Beliefs about capabilities | *Willingness to act* | Persuasion  Education  Modelling | Communication/ Marketing | 9.1 Credible source  15.1 Verbal persuasion about capability  15.2 Mental rehearsal of successful performance  15.3 Focus of past success  15.4 Self-talk |
|  | Intentions | *Willingness to act* | Education  Persuasion  Incentivisation  Modelling | Communication/ Marketing  Guidelines | - 1. Goal setting (behaviour)   1.3 Goal setting (outcome)  9.1 Credible source  10.8 Incentive (outcome) |
|  | Beliefs about consequences  Reinforcement | *Necessity and concerns* | Education  Persuasion  Modelling | Communication/ Marketing  Environmental/ Social planning | 2.7 Feedback on outcome(s) of behaviour  5.1 Information about health consequences  5.2 Salience of consequences  5.3 Information about social/ environmental consequences  5.6 Information about emotional consequences  9.1 Credible source  13.2 Framing/ reframing |
|  | Goals | *Necessity and concerns* | Education  Persuasion  Incentivisation  Modelling | Communication/ Marketing  Guidelines  Environmental/ Social planning  Service provision | - 1. Goal setting (Behaviour)   1.3 Goal setting (outcome)  5.1 Information about health consequences  5.2 Salience of consequences  5.3 Information about social/environmental consequences  5.6 Information about emotional consequences  13.2 Framing/reframing |
|  | Optimism | *Necessity and concerns* | Education  Persuasion  Modelling | Communication/ Marketing  Guidelines | 5.1 Information about health consequences  5.2 Salience of consequences  5.3 Information about social/environmental consequences  5.6 Information about emotional consequences |
| Motivation (Automatic) | Emotion | *Emotional Impact* | Persuasion  Incentivisation  Modelling  Enablement  Coercion | Communication/ Marketing  Environmental/ Social planning  Service provision  Guidelines | 1.2 Problem solving  1.4 Action planning  3.1 Social support (unspecified)  3.3 Social support (emotional)  5.6 Information about emotional consequences  11.2 Reduce negative emotions  11.3 Conserving mental resources  13.2 Framing/reframing |
